# Editorial: Advances in systemic lupus erythematosus: The promises and pitfalls of personalized medicine and patient stratification

**DOI:** 10.3389/fmed.2022.1085719

**Published:** 2022-12-06

**Authors:** Emiliano Marasco, Johanna Mucke, Jolien Suurmond

**Affiliations:** ^1^Department of Rheumatology, Bambino Gesù Children's Hospital, Rome, Italy; ^2^Department of Rheumatology, University Hospital Düsseldorf, Medical Faculty of Heinrich Heine University, Düsseldorf, Germany; ^3^Hiller Research Center, University Hospital Düsseldorf, Medical Faculty of Heinrich Heine University, Düsseldorf, Germany; ^4^Department of Rheumatology, Leiden University Medical Center (LUMC), Leiden, Netherlands

**Keywords:** systemic lupus erythematosus, personalized medicine, biomarker, autoantibodies, mortality, Thrombotic Microangiopathy, macrophage activation syndrome (MAS)

Systemic lupus erythematosus (SLE) is a complex autoimmune disease where multiple pathways play a role in its pathogenesis (1). The biological heterogeneity of SLE reflects a clinical heterogeneity with diverse clinical manifestations ranging from constitutional symptoms to organ-threatening diseases such as renal or CNS involvement. Patient stratification for prognosis (i.e., future development of organ involvement) and tailored treatment could be a game-changer in the management of SLE. Despite extensive efforts into developing such stratifications, we are still far from having robust biomarkers implemented in everyday practice.

An area of research that is under-addressed and lacks robust and consistent studies is rare manifestations of SLE. In these scenarios, diagnosis and treatment can be challenging. The rarity of these manifestations is an intrinsic limit that hinders their understanding. Thrombotic Microangiopathy (TMA) is characterized by microvascular occlusion in association with thrombocytopenia and microangiopathic hemolytic anemia, leading to severe organ injury, mainly kidney failure. Yang et al. describe the clinical features and outcome of patients with SLE and identified thrombocytopenia as an independent risk factor for mortality.

Macrophage activation syndrome (MAS) is a complication of rheumatic diseases, most frequently systemic Juvenile Idiopathic Arthritis (sJIA), but also SLE. Abdirakhmanova et al. performed a systematic review of the literature, confirming that MAS complicating SLE poses a significant challenge to clinicians with delayed diagnosis and treatment. Furthermore, the authors assessed the performance of the classification criteria for MAS validated in sJIA patients and the preliminary diagnostic guidelines for MAS in pediatric SLE. The preliminary diagnostic guidelines appeared to outperform the MAS classification criteria for sJIA in SLE, highlighting the importance to develop and validate new criteria specific for SLE.

SLE poses a significant health burden on patients. The 10-year survival rate for patients with SLE improved from 63.2% in the 1950s to 91.4% in the 2000s (2). However, mortality is still higher than that of the general population. Patients are frequently hospitalized for either active disease, disease damage and comorbidities or side effects from medications (i.e., infections). Particularly, admission to the intensive care unit (ICU) is burdened by significant mortality. Guo et al. analyzed the data of patients with SLE admitted to the ICU and identified 9 factors that could predict mortality. The authors built a prognostic model to predict mortality in patients admitted to the ICU, which could help to stratify patients and identify those at high risk requiring a more intensive management.

Although, several biomarkers for patients' stratification, follow-up monitoring and tailored comorbidity screenings have been investigated, none have yet been implemented in everyday practice. Further studies on predictive biomarkers are thus essential.

In contrast to the growing number of new treatments approved every year for rheumatoid arthritis (RA), SLE has seen only a few drugs receiving approval by agencies, with belimumab approved around 12 years ago (3), and more recently with the approval of anifrolumab and voclosporin. Rituximab (RTX) is a chimeric antibody that targets CD20 expressed on B cells, inducing B cell depletion. Although two phase II/III trials of RTX in SLE did not meet the primary endpoint (4, 5), RTX is routinely used in clinical practice to treat SLE, especially the most refractory cases. RTX has several well-known side effects, from increased infection risk to infusion reactions. Gilaberte Reyzabal and Isenberg reviewed the occurrence of adverse infusion reactions to RTX in SLE and highlighted that these were less than what was observed in patients with non-Hodgkin lymphoma. The authors propose that the more frequent use of corticosteroids in patients SLE is responsible for the low frequency of infusion reaction to RTX. However, the exact mechanisms have not been identified yet.

It is evident that a better understating of the biology of SLE is required in order to provide the grounds for implementing new biomarkers for patient stratification in SLE and developing new treatment strategies.

The current Research Topic presents several studies proposing novel biomarkers for patients with SLE to predict mortality and the occurrence of rare disease manifestations. We hope this research will spark new and exciting studies to better understand the biology of SLE and how to stratify patients.

## Author contributions

All authors listed have made a substantial, direct, and intellectual contribution to the work and approved it for publication.
